# Anesthesiological Management of a Patient with Williams Syndrome Undergoing Spine Surgery

**DOI:** 10.1155/2016/1371095

**Published:** 2016-03-16

**Authors:** Federico Boncagni, Luca Pecora, Vasco Durazzi, Francesco Ventrella

**Affiliations:** ^1^Clinica di Rianimazione Generale, Respiratoria e del Trauma Maggiore, Azienda Ospedaliero-Universitaria “Ospedali Riuniti Umberto I-G. M. Lancisi-G. Salesi”, 60126 Ancona, Italy; ^2^Anestesia e Rianimazione dei Trapianti e della Chirurgia Maggiore, Azienda Ospedaliero-Universitaria “Ospedali Riuniti Umberto I-G. M. Lancisi-G. Salesi”, 60126 Ancona, Italy; ^3^Clinica di Neurologia, Azienda Ospedaliero-Universitaria “Ospedali Riuniti Umberto I-G. M. Lancisi-G. Salesi”, 60126 Ancona, Italy; ^4^Anestesia e Rianimazione Pediatrica, Azienda Ospedaliero-Universitaria “Ospedali Riuniti Umberto I-G. M. Lancisi-G. Salesi”, 60126 Ancona, Italy

## Abstract

Williams Syndrome (WS) is a complex neurodevelopmental disorder associated with a mutation on chromosome 7. Patients with WS usually display dysmorphic facial and musculoskeletal features, congenital heart diseases, metabolic disturbances and cognitive impairment. Structural cardiovascular abnormalities are present in the majority of the children and may provide a substrate for perioperative Sudden Cardiac Death, as presented by several reports, something that creates a great challenge to the anesthetic conduct. We present the case of a 12-year old girl who required anesthetic care for surgical correction of an acquired kyphoscoliosis. Potential anesthesiological implications of WS are subsequently reviewed.

## 1. Introduction

Williams-Beuren Syndrome (WBS) is a genetic autosomal dominant disorder associated with* de novo* deletion in the long arm of chromosome 7 (7q11.23), occurring 1 : 10.000 live births [1–3]. Patients with WBS show mild to moderate mental retardation and musculoskeletal and craniofacial abnormalities, such as hypertelorism, flat nasal bridge, long philtrum, and wide mouth with a hypoplastic or short mandible [1, 3]. Considering the association with congenital heart defects, Supravalvular Aortic Stenosis (SVAS) and Pulmonary Artery Stenosis (PAS) are reported in up to 80% of pediatric patients [2], clinical conditions that create enormous difficulty in the anesthetic approach, something that can be translated by the high probability of refractory intraoperative cardiac arrest [4–8]. Given to the multiorgan involvement of the syndrome and to the anecdotal nature of case descriptions, anesthesiological management of affected patients is often challenging and it implies special considerations to cardiovascular, metabolic, and technical aspects of anesthesia delivering. In this case report, we describe the anesthetic management of a 12-year-old girl undergoing anesthesia for surgical correction of an acquired kyphoscoliosis and we subsequently discuss perioperative implications of WBS.

Written informed consent from patient's relatives was obtained before case report publication and it is available on editors' request.

## 2. Case Presentation

A 12-year-old girl with WBS was referred to our centre for definitive correction of a developmental, thoracic kyphoscoliosis. She already underwent the provisional placement of a posterior extensible distractor three years ago. The diagnosis of WBS, based on* stellata iris *pattern of the eyes, umbilical hernia, interatrial septal defect, and failure to thrive, was presumed at 7 months by her paediatrician and further confirmed by genetic analysis (FISH hybridization) which showed the typical hemizygosity at 7q11.23.

During the follow-up, a spontaneous closure of cardiac septal defect, hypercalcemia, controlled by dietary interventions, repeated episodes of fever of unknown origin by one year, and occurrence of Schönlein-Henoch purpura by two months, both, before admission to surgery were found.

**Figure 1:**
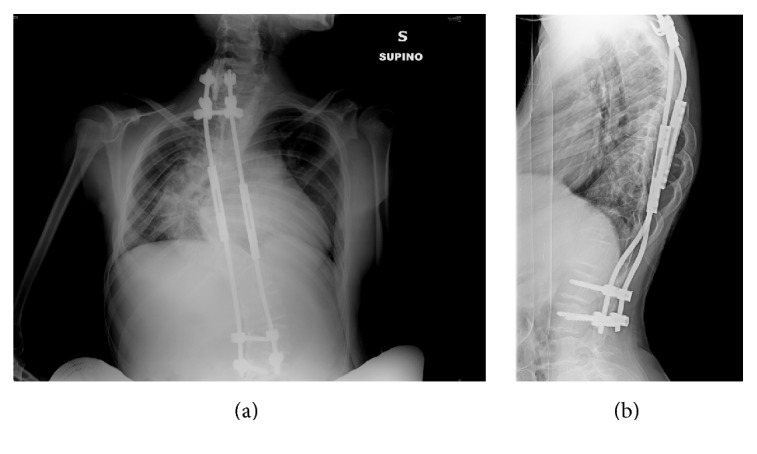
Anteroposterior (a) and lateral (b) chest X-ray showing patient's pronounced spinal curvature and dorsal hump. A provisional extensible distractor is in place.

Physical examination showed the characteristic appearance of WBS patients with broad forehead, flat nasal bridge, wide mouth with a prominent lower lip, dental malocclusion, and a short mandible. Spinal curvature was more than 80° in the coronal plane with an associated dorsal hump (Figure 1). She weighed 35 Kg and her vital signs were within the limits, with blood pressure (BP) of 130/90 mmHg. Baseline ECG showed sinus rhythm at 88 beats/min with a normal QRS and no ST-T alterations. Cardiopulmonary physical examination only showed slight 2/6 systodiastolic murmur at the precordium. Preoperative laboratory exams included TSH, thyroid hormones, and calcium, which were within normal range. A transthoracic echocardiogram, performed to rule out previously unrecognized cardiac abnormalities, showed a nonsignificant interventricular septal defect, absence of SVAS, or interatrial septal defect. Color-Doppler examination of supra-aortic trunks showed no thickening of the carotids. Given that the patient was asymptomatic under the cardiovascular point of view and doing well with everyday activities, we chose not to perform a coronary angiography.

**Figure 2:**
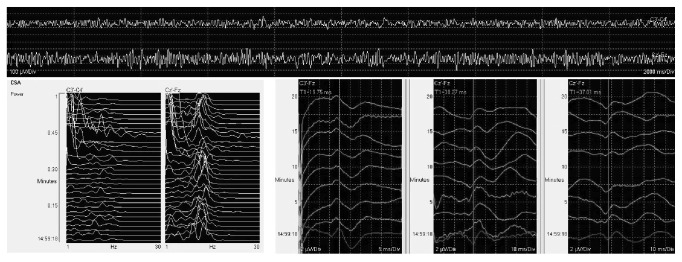
Screenshot of continuous EEG, spectral EEG analysis, and SSEPs (somatosensory evoked potentials) at the end of the procedure. EEG monitoring under general anesthesia shows persistence of *α*-like activity (top) with a peak frequency around 9-10 Hz (left). Right corner of the image: SSEPs monitoring is shown. Physiological neuraxial propagation of peripheral stimuli (darker line) with no time delay is seen (from left to right: the first column is right upper limb channel, and then the right lower limb and the left lower limb channels are shown, second and third column, resp.).

A 4.5-French central venous catheter was placed in the right internal jugular vein under light sedation and local anesthesia, in the day before the surgical procedure. The child was kept quiet for eight hours and transported to the operating room the day of the surgery. Atropine 0.4 mg was given intravenously soon before anesthesia induction with fentanyl 100 mcg and propofol 120 mg. No neuromuscular blocking agent was used. Prior to intubation, the glottis was irrigated under direct laryngoscopy with 10 mL of 2% lidocaine via a Optispray® syringe, revealing grade I Cormack-Lehane visualization of vocal cords. Tracheal intubation was achieved at first attempt after local anesthesia with a 6.5 mm ID cuffed tube. After intubation, left radial artery was cannulated and a urinary catheter was placed. Finally, the patient was prone positioned. The maintenance of the anesthesia consisted of 2% sevoflurane in O_2_/air mixture and remifentanil continuous infusion. Intraoperative monitoring included pulse oximetry, continuous ECG tracing, invasive blood pressure, and hemodynamic monitoring via a FloTrac/Vigileo® device, end tidal carbon dioxide, and bladder temperature. Neurophysiological monitoring was provided by continuous EEG and somatosensory evoked potentials (SSEPs) to detect spinal lesions occurring during surgery. We attempted to maintain blood pressure of 90/50 mmHg throughout the whole procedure, with an average cardiac index (CI) around 4.5 L/min/m^2^. Minimum bladder temperature at the end of the surgery was 35°C. No alteration of SSEPs propagation compared to baseline was noted (Figure 2). Total surgical time was 7 h 16 min, with an estimated blood loss of 500 mL. In turn, during the surgery, it was observed that the correction of the original kyphoscoliosis was impossible, due to excessive rigidity of the spine. Therefore, only definitive spinal fusion was performed. After supine repositioning, the maintenance of anesthesia was stopped and the patient extubated, without complications. Clinical observation continued for about 30 min in the operating theatre, and then the child was moved to the department of orthopaedics. Analgesia was provided by means of continuous infusion of morphine 20 mg, ketorolac 30 mg, and ketamine 100 mg, over the following 24 hours. The postoperative course was uneventful and the child was discharged home on day 8.

## 3. Discussion

Williams-Beuren Syndrome (WBS), originally first described by Williams et al. in 1961 [9], is a congenital disease that occurs due to deletion in the long arm of chromosome 7. Typically, the deletion involves a region spanning 1.5 to 1.8 Mb that encodes for several genes, including the elastin (ELN) gene, and it occurs during meiotic crossover as a result of chromosome misalignment [1, 10]. Either the maternally or the paternally inherited chromosome 7 may be involved, the phenomenon being sporadic in the majority of the cases. Elastin deficiency and defective expression of contiguous genes have been regarded, as possible explanations of the multiorgan involvement of the disease [1, 10, 11]. Typically, affected individuals display a variable degree of mental retardation (mean IQ range 41–80), a friendly and hypersocial personality with characteristic facial stigmata, such as epicanthal folds, wide-set eyes, flat nasal bridge, a prominent lower lip, and puffy cheeks (“elfin face”). The mandible is often short or hypoplastic. Blue-eyed patients may have a “stellate” pattern of their irides (*stellata iris*) [12].

Pediatric patients may turn to anesthesiologists early in their lives, for surgical repair of cardiovascular defects, multiple hernias of the abdominal wall, and ENT procedures [8, 12, 13]. Anesthesiological management is often challenging and implies in careful preoperative evaluation, aiming to exclude potential comorbidities.

Supravalvular Aortic Stenosis (SVAS) is the most common congenital cardiac defect encountered in children with WBS. It may appear as hourglass narrowing, at the sinotubular junction, or as diffuse stenosis of the ascending aorta, involving the brachiocephalic vessels [3, 9, 14], with frequently stenotic and tortuous coronary arteries [15]. Cardiac arrest under general anesthesia has been reported in several case series, likely as a result of a perfusion mismatch in the presence of a severe left ventricular outflow tract (LVOT) obstruction [4–8]. Since WBS clinical presentation is variable, less severe forms of cardiac defects are possible, as in our case. A detailed review of previous medical history and a preoperative work-up, including at least 4-limb blood pressure measurement, ECG, and a transthoracic echocardiogram screening, will help the identification of high-risk of adverse events, which serve as reference for the care to be adopted by the teams. Although questionable, a coronary angiography has been recommended by some authors for any WBS patient requiring anesthetic care [5]. Besides congenital heart diseases, elastin gene knockout leads to diffuse arteriopathy: specific lesions imply a thickening of supra-aortic trunks [16] and renovascular involvement [17, 18]. Actually, early-onset hypertension is a common finding among affected patients [19–21].

The presence of a difficult airway may be a reason for concern, too. Despite the fact that clinical reports are substantially lacking, distinct anatomic features of WBS patients are predictors of both difficult intubation and bag-mask ventilation, especially mandible hypoplasia, dental abnormalities, and flattened maxillary profile [13, 22]. It is not clearly established whether WBS patients are at higher risk for airway obstruction. Nonetheless, it has been inferred that elastin deficiency may affect vocal cords function, thereby providing an explanation for WBS individuals' hoarse voice. There are at least two descriptions of laryngeal stridor; one of these is occurring in the postoperative setting and successfully managed with inhaled epinephrine [13, 23]. Joint laxity and muscle weakness are commonly reported in WBS, and a variable degree of myopathy has been demonstrated. Caution has been advocated in the use of neuromuscular blocking agents (NMBAs), since the risk of a prolonged response could not be excluded [13, 24].

Endocrine anomalies are commonly represented by hypercalcemia and hypothyroidism. Infantile hypercalcemia has been reported in up to 15% of pediatric patients and usually resolves in the first years of life. It is generally mild and rarely accompanied by nephrocalcinosis. However, calcium imbalances may be recurrent during puberty and preoperative screening is recommended [12, 25]. Subclinical hypothyroidism has been described in 15 to 30% of WBS screened patients. Although overt hypothyroidism is a rare finding, clinical implications on cardiac function, blood volume, perioperative anemia, and temperature homeostasis prompt preoperative thyroid function evaluation [26, 27].

In summary, anesthetic care of WBS patients demands particular attention. The identification of cardiovascular comorbidities is the most challenging task and meticulous preoperative evaluation of heart defects should be warranted. However, anesthesiologists should be aware of the fact that the management of these patients should address all the multisystemic aspects of this complex syndrome.
